# Mental health status at initial counseling sessions among US university students: A comparative analysis by race and religion

**DOI:** 10.1002/pcn5.70209

**Published:** 2025-09-28

**Authors:** Nicole Nagib, Ryo Horita, Natalie Nagib, Taku Fukao, Nanako Imamura, Satoko Tajirika, Miho Adachi, Minako Kawamoto, Mayumi Yamamoto

**Affiliations:** ^1^ College of Public Health University of South Florida Tampa Florida USA; ^2^ Health Administration Center Gifu University Gifu Gifu Japan; ^3^ Psychiatry Gifu University Hospital Gifu Gifu Japan; ^4^ Medical Education Development Center Gifu University Gifu Gifu Japan; ^5^ United Graduate School of Drug Discovery and Medical Information Sciences Gifu University Gifu Gifu Japan; ^6^ College of Medicine Lake Erie College of Osteopathic Medicine Bradenton Florida USA

**Keywords:** Counseling Center Assessment of Psychological Symptoms, mental health, psychological distress, race, religion

## Abstract

**Aim:**

We analyzed university students' mental health to inform culturally tailored interventions within university counseling services, focusing on race and religion as key variables.

**Methods:**

Data were collected over 5 years from students at X University attending their first counseling session (*N* = 9373). Participants completed the Counseling Center Assessment of Psychological Symptoms (CCAPS). Differences in CCAPS scores were examined using a one‐way multivariate analysis of variance.

**Results:**

Asian American students had high depression and hostility scores, whereas White students had lower distress scores. Asian American students scored the highest in potential thought disturbance and suicidal thoughts. Hindu and Jewish students scored the highest and lowest in depression, respectively.

**Conclusion:**

University students' mental health varies significantly by race and religion, underscoring the importance of culturally competent interventions within university counseling centers. This highlights the need for implementing targeted and tailored mental health strategies among diverse student populations.

## INTRODUCTION

University life represents a pivotal transition period characterized by significant developmental changes and heightened stressors, making the mental health of university students a critical area of focus. Recent studies indicate an increasing prevalence of psychological distress among students,^1^ often attributed to academic pressures and social adjustments.^2^ University counseling centers widely use tools such as the Counseling Center Assessment of Psychological Symptoms (CCAPS) to evaluate these challenges, covering a broad spectrum of symptoms, including depression, anxiety, academic distress, and social concerns.^3^

Although existing research highlights various factors contributing to mental health disparities among university students, a deeper examination of intersecting identities such as race and religion remains limited. Racial and ethnic minority students often report unique stressors, such as discrimination and cultural adaptation issues, which significantly impact their psychological well‐being.^4^ Students from these backgrounds may have undetected psychiatric problems and, therefore, represent a particularly at‐risk group on campus.^5^ Concurrently, religious identity serves as both a protective factor and a potential stressor depending on the context, thereby influencing mental health outcomes differently across groups.^6^

The interplay between race and religion introduces a nuanced dimension to understanding mental health disparities. Intersectionality theory posits that overlapping social identities and systemic inequalities compound these effects, with minority groups often experiencing heightened vulnerability.^7^ For university counseling services, recognizing and addressing these intersections is critical to developing culturally competent interventions.

This study analyzes the CCAPS scores of university students to examine how race and religious identity intersect to influence mental health outcomes. There are nationwide surveys targeting universities in the United States^8^; however, research specifically focusing on the mental health of students who have used counseling services remains insufficiently accumulated. By doing so, it aims to inform strategies for culturally sensitive support systems within campus counseling services, addressing a gap in the literature regarding the mental health experiences of racially and religiously diverse student populations.

## METHODS

### Study design

This study was conducted over a 5‐year period (2019–2023). It adhered to the Strengthening the Reporting of Observational Studies in Epidemiology guidelines.^9^ Participants were students attending their first counseling session at X University, a public research university in the United States.

### Participants and procedures

An online survey^3^ was administered before the first counseling appointment with student counselors. The inclusion criteria were as follows: (1) first‐time counseling visit at X University during the academic years 2019, 2020, 2021, 2022, or 2023, (2) completion of the CCAPS questionnaire, and (3) responses to the demographic questions regarding race/ethnicity and religion. A total of 9373 students were included in the study sample. Table 1 shows the breakdown by race/ethnicity and religion.

**Table 1:** Sample demographics and characteristics.

Variables	Total sample	
	*n*	%
Total participants	9373	100
Race/ethnicity		
African American	1010	10.77
Asian American/Asian	1185	12.64
Hispanic/Latino(a)	1805	19.26
Multiracial	628	6.70
White	4745	50.62
Religion		
Agnostic	1601	17.08
Atheist	874	9.32
Buddhist	107	1.14
Catholic	1132	12.08
Christian	2419	25.81
Hindu	242	2.58
Jewish	187	2.00
Muslim	323	3.45
No preference	2488	26.54

### Measure

The CCAPS^10^ is a self‐report instrument designed to evaluate the psychological symptoms of university students. It serves as a comprehensive tool for assessing a range of mental health concerns frequently observed in college populations.^3^ The CCAPS contains 62 items categorized into eight subscales: depression, generalized anxiety, social anxiety, academic distress, eating concerns, hostility, family distress, and substance use. Additionally, the CCAPS includes four critical items that assess risk factors such as potential thought disturbance (I lose touch with reality), suicidal ideation (I have thoughts of ending my life), violent behavior (I am afraid I may lose control and act violently), and homicidal ideation (I have thoughts of hurting others). Responses are measured on a five‐point Likert scale from 0 (*not at all like me*) to 4 (*extremely like me*), with higher scores reflecting greater levels of psychological distress. The CCAPS has demonstrated strong psychometric properties, including robust internal consistency, good test‐retest reliability, and valid factor structures across different university settings.

### Data analysis

This study examined the eight subscales and four critical items of the CCAPS in relation to students' reported race and religion. A cross‐sectional analysis was conducted to compare scores across the eight subscales and four critical items based on demographic variables. Two separate one‐way multivariate analysis of variance (MANOVA) were conducted to explore the relationships between the independent variables (race and religion, respectively) and the 12 dependent variables (the eight subscales and four critical items). Effect sizes were measured using partial eta‐squared values, with thresholds of 0 to <0.06 for a small effect, 0.06 to <0.14 for a moderate effect, and ≥0.14 for a large effect. Statistical significance was set at *p* < 0.05. Data analysis was performed using spss version 28.0 (IBM, Armonk, NY, USA).

### Ethical considerations

All study procedures complied with the ethical standards set by national and institutional committees overseeing human research and were consistent with the principles of the 1964 Declaration of Helsinki and its later revisions. The authors have no conflicts of interest to report, and the study design was approved by the ethical review committee of the Graduate School of Medicine, Gifu University, Japan (no. 2021‐B114). Additionally, we obtained consent from X University, where the data were collected for the provision procedures.

## RESULTS

### Race/ethnicity

The results of the one‐way MANOVA and post hoc analysis by race are summarized in Table 2. The one‐way MANOVA revealed a significant relationship between race and the seven subscales (excluding social anxiety) as well as all four critical items. Asian American students consistently had the highest scores for depression, academic distress, eating concerns, hostility, and critical items (excluding homicidal ideation). Similarly, multiracial students had the highest scores in generalized anxiety, social anxiety, and family distress. However, the eta‐squared statistic reflecting effect size indicated no effect in all subscales and critical items except for violent behavior, *F*(4, 9368) = 36.37, *p* < 0.001, *η* ^2^ = 0.015.

**Table 2:** Average Counseling Center Assessment Psychological Symptoms (CCAPS) scores by race/ethnicity.

	1: African American *M* (SD)	2: Asian American *M* (SD)	3: Hispanic/Latino(a) *M* (SD)	4: Multiracial *M* (SD)	5: White *M* (SD)	*F*	Effect size *η* ^2^	Post hoc Tukey HSD
Average CCAPS score								
Depression	1.97 (0.90)	2.11 (0.88)	1.93 (0.88)	2.04 (0.89)	1.91 (0.87)	13.73***	0.006	1 < 2, 3 < 2, 5 < 2, 5 < 4
Generalized anxiety	1.88 (0.93)	1.98 (0.91)	1.99 (0.92)	2.03 (0.90)	2.03 (0.90)	5.90***	0.003	1 < 3, 1 < 4, 1 < 5
Social anxiety	2.17 (0.74)	2.22 (0.73)	2.18 (0.76)	2.24 (0.77)	2.21 (0.74)	2.03	0.001	
Academic distress	2.10 (1.00)	2.15 (0.99)	2.08 (1.01)	2.12 (1.04)	1.99 (0.99)	9.13***	0.004	5 < 1, 5 < 2, 5 < 3, 5 < 4
Eating concerns	1.34 (0.75)	1.47 (0.78)	1.42 (0.80)	1.41 (0.79)	1.40 (0.78)	3.97**	0.002	1 < 2
Hostility	1.08 (0.84)	1.24 (0.90)	1.11 (0.87)	1.15 (0.85)	1.04 (0.81)	14.70***	0.006	1 < 2, 3 < 2, 5 < 2, 5 < 3, 5 < 4
Family distress	1.65 (0.98)	1.67 (1.01)	1.55 (1.01)	1.79 (1.04)	1.51 (1.01)	16.55***	0.007	5 < 1, 5 < 2, 3 < 2, 3 < 4, 5 < 4
Substance use	0.50 (0.74)	0.47 (0.70)	0.55 (0.78)	0.60 (0.75)	0.62 (0.79)	12.31***	0.005	1 < 5, 2 < 4, 2 < 5, 3 < 5
Critical items								
Potential thought disturbance	1.57 (1.44)	1.75 (1.41)	1.55 (1.43)	1.65 (1.40)	1.50 (1.38)	8.83***	0.004	1 < 2, 5 < 2, 2 < 3
Suicidal ideation	0.90 (1.26)	1.00 (1.29)	0.74 (1.11)	0.92 (1.26)	0.70 (1.10)	21.54***	0.009	3 < 1, 5 < 1, 3 < 2, 5 < 2, 3 < 4, 5 < 4
Violent behavior	0.54 (1.00)	0.73 (1.22)	0.47 (0.97)	0.48 (0.96)	0.37 (0.86)	36.37***	0.015	5 < 1, 1 < 2, 3 < 2, 4 < 2, 5 < 2, 5 < 3
Homicidal ideation	0.23 (0.67)	0.22 (0.65)	0.13 (0.50)	0.24 (0.66)	0.14 (0.52)	12.61***	0.005	3 < 1, 3 < 2, 3 < 4, 5 < 1, 5 < 2, 5 < 4

*Note*: Multivariate analysis of variance test.

Abbreviations: HSD, honestly significant difference; SD, standard deviation

** *p* < 0.01,

*** *p* < 0.001.

### Religious identity

The results of the one‐way MANOVA and post hoc analysis by religion are summarized in Table 3. The one‐way MANOVA revealed a significant relationship between religion and all eight subscales, as well as all four critical items. Hindu students had the highest scores in depression, eating concerns, and hostility. Buddhist students had the highest scores for generalized anxiety, academic distress, and family distress. A small effect size was observed in depression, *F*(8, 9364) = 14.78, *p* < 0.001, *η* ^2^ = 0.012; family distress, *F*(8, 9364) = 23.95, *p* < 0.001, *η* ^2^ = 0.020; substance use, *F*(8, 9364) = 13.40, *p* < 0.001, *η* ^2^ = 0.011; and suicidal ideation, *F*(8, 9364) = 15.43, *p* < 0.001, *η* ^2^ = 0.013.

**Table 3:** Average Counseling Center Assessment Psychological Symptoms (CCAPS) scores by religious identity.

	1: Agnostic *M* (SD*)*	2: Atheist *M* (SD)	3: Buddhist *M* (SD)	4: Catholic *M* (SD)	5: Christian *M* (SD)	6: Hindu *M* (SD)	7: Jewish *M* (SD)	8: Muslim *M* (SD)	9: No preference *M* (SD)	*F*	Effect size *η* ^2^	Post hoc Tukey HSD
Average CCAPS score												
Depression	2.02 (0.87)	2.02 (0.86)	2.06 (0.88)	1.88 (0.88)	1.84 (0.87)	2.19 (0.90)	1.67 (0.82)	2.13 (0.89)	2.00 (0.89)	14.78***	0.012	4 < 1, 5 < 1, 7 < 1, 4 < 2, 5 < 2, 7 < 2, 7 < 3, 4 < 6, 4 < 8, 4 < 9, 5 < 6, 5 < 8, 5 < 9, 7 < 6, 9 < 6, 7 < 8, 9 < 8
Generalized anxiety	2.05 (0.90)	2.00 (0.88)	2.12 (0.94)	1.95 (0.93)	1.92 (0.90)	2.10 (0.95)	2.00 (0.91)	2.09 (0.89)	2.03 (0.92)	4.82***	0.004	5 < 1, 5 < 9
Social anxiety	2.27 (0.73)	2.31 (0.75)	2.26 (0.75)	2.15 (0.74)	2.13 (0.74)	2.19 (0.76)	2.04 (0.73)	2.19 (0.73)	2.24 (0.74)	9.81***	0.008	4 < 1, 5 < 1, 4 < 2, 5 < 2, 7 < 2, 4 < 9, 5 < 9, 7 < 9, 7 < 1,7 < 2
Academic distress	2.10 (1.00)	2.09 (0.97)	2.26 (0.97)	1.92 (1.00)	1.99 (1.00)	2.09 (0.99)	1.77 (0.99)	2.17 (1.02)	2.09 (1.01)	7.66***	0.007	4 < 1, 7 < 1, 4 < 2, 7 < 2, 4 < 3, 7 < 3, 4 < 8, 4 < 9, 5 < 9, 5 < 1, 7 < 6, 3 < 7, 7 < 8, 7 < 9
Eating concerns	1.44 (0.79)	1.42 (0.81)	1.54 (0.76)	1.41 (0.79)	1.36 (0.76)	1.59 (0.86)	1.46 (0.77)	1.48 (0.78)	1.40 (0.77)	4.26***	0.004	5 < 1, 4 < 6, 5 < 6, 9 < 6
Hostility	1.11 (0.81)	1.12 (0.84)	1.15 (0.90)	1.08 (0.88)	1.01 (0.80)	1.35 (0.90)	0.87 (0.79)	1.34 (0.91)	1.10 (0.84)	11.21***	0.009	5 < 1, 1 < 6, 7 < 1, 1 < 8, 5 < 2, 2 < 6, 7 < 2, 2 < 8, 9 < 6, 4 < 6, 4 < 8, 5 < 6, 5 < 8, 7 < 6, 7 < 9, 7 < 8, 9 < 8, 5 < 9
Family distress	1.75 (1.05)	1.67 (1.01)	1.99 (0.94)	1.34 (0.96)	1.47 (0.99)	1.51 (1.01)	1.31 (1.00)	1.66 (1.01)	1.63 (1.01)	23.95***	0.020	4 < 1, 5 < 1, 6 < 1, 7 < 1, 4 < 2, 5 < 2, 7 < 2, 4 < 3, 5 < 3, 6 < 3, 7 < 3, 9 < 3, 4 < 5, 4 < 9, 5 < 9, 4 < 8, 5 < 8, 7 < 8, 9 < 1, 7 < 9
Substance use	0.68 (0.85)	0.65 (0.81)	0.53 (0.75)	0.58 (0.77)	0.50 (0.72)	0.53 (0.75)	0.61 (0.76)	0.31 (0.64)	0.59 (0.75)	13.40***	0.011	4 < 1, 5 < 1, 8 < 1, 5 < 2, 8 < 2, 8 < 4, 8 < 5, 8 < 6, 8 < 7, 8 < 9, 5 < 9, 9 < 1
Critical items												
Potential thought disturbance	1.66 (1.40)	1.49 (1.38)	1.79 (1.48)	1.45 (1.39)	1.42 (1.39)	1.82 (1.48)	1.33 (1.35)	1.80 (1.42)	1.64 (1.42)	9.25***	0.008	4 < 1, 5 < 1, 4 < 6, 2 < 8, 4 < 8, 5 < 6, 5 < 8, 7 < 6, 7 < 8, 4 < 9, 5 < 9
Suicidal ideation	0.91 (1.20)	0.90 (1.23)	0.93 (1.22)	0.64 (1.09)	0.63 (1.06)	0.95 (1.32)	0.42 (.85)	0.83 (1.23)	0.86 (1.21)	15.43***	0.013	4 < 1, 5 < 1, 7 < 1, 4 < 2, 5 < 2, 7 < 2, 4 < 6, 7 < 6, 4 < 9, 5 < 9, 7 < 9, 7 < 3, 5 < 6, 7 < 8
Violent behavior	0.43 (0.92)	0.43 (0.91)	0.71 (1.17)	0.45 (0.97)	0.40 (0.90)	0.77 (1.26)	0.33 ((.80)	0.78 (1.22)	0.48 (0.97)	10.62***	0.009	1 < 6, 1 < 8, 2 < 6, 2 < 8, 3 < 5, 3 < 7, 4 < 6, 4 < 8, 5 < 6, 5 < 8, 7 < 6, 7 < 8, 9 < 6, 9 < 8
Homicidal ideation	0.16 (0.56)	0.19 (0.61)	0.18 (0.56)	0.14 (0.55)	0.14 (0.52)	0.12 (0.45)	0.17 (0.59)	0.25 (0.67)	0.18 (0.58)	2.26*	0.002	5 < 8

*Note*: Multivariate analysis of variance test.

Abbreviations: HSD, honestly significant difference; SD, standard deviation.

* *p* < 0.05

*** *p* < 0.001.

## DISCUSSION

This study investigated the impact of race and religious identity on mental health outcomes among university students who visit counseling centers, as measured by the CCAPS. The results highlighted significant variations in mental health symptoms across different racial and religious groups, providing valuable insights into the social determinants that influence psychological well‐being in diverse student populations.

### Influence of race on mental health

The results revealed significant differences in mental health symptoms across racial groups, emphasizing the importance of considering race when assessing and addressing mental health needs. The finding that Asian American and multiracial students had poorer mental health compared to other racial groups is consistent with previous research suggesting that cultural stigma surrounding mental health and the “model minority” stereotype exacerbate distress in this population.^11^ When supporting students with roots in these ethnic groups, more detailed and attentive support is required. The elevated levels of family distress may also reflect pressures related to intergenerational expectations and cultural values. Conversely, White students reported the lowest levels of distress across most domains, including depression, academic distress, hostility, and family distress. These findings suggest that systemic advantages and reduced exposure to discrimination may contribute to better mental health outcomes in this group.

### Role of religious identity in mental health

Religious identity emerged as a significant factor in shaping mental health outcomes. The finding that Hindu students had poorer mental health compared to other religious groups highlights the potential stressors associated with navigating religious practices in secular or predominantly non‐Hindu environments.^12^ Further, the elevated family distress reported by Buddhist students may reflect cultural expectations and familial obligations that compound stress. In contrast, Jewish students consistently reported the lowest levels of depression, social anxiety, academic distress, hostility, family distress, and critical items (excluding homicidal ideation). This finding may be attributable to strong community support and cultural resilience, which can buffer against mental health challenges.^13^ Many of the substance use factor items are related to alcohol consumption. Therefore, the scores of Muslim students, for whom alcohol is prohibited, were significantly lower. Additionally, both agnostic and atheist students demonstrated statistically significant differences in several mental health domains compared to students in other religious groups. Although neither group reports formal religious affiliation, they differ in their perspectives on belief: atheists typically reject the existence of deities, whereas agnostics regard the existence or nonexistence of deities as unknown or unknowable. These differences may influence coping strategies, social networks, and perceived community support. The relatively higher scores observed in certain symptom domains among these groups may reflect the absence of protective factors often associated with organized religion, such as communal support or shared coping resources. However, further research is warranted to explore whether these differences are attributable to worldview, social connectedness, or other unmeasured factors. The differences across religious groups underscore the complex interplay between faith, community dynamics, and mental health.

### Implications for mental health practice

The findings underscore the need for targeted mental health interventions that are culturally sensitive and inclusive of diverse racial and religious identities.^14^ Universities and mental health professionals should consider these identities when developing support systems and resources, ensuring that services are tailored to meet the unique needs of each group. By recognizing and addressing the diverse experiences within and across different racial and religious identities, we can create more effective and equitable mental health support for all students.

Additionally, although statistically significant differences were observed in many comparisons, the effect sizes (partial eta‐squared) were in the small range. Given the large sample size, even small differences can reach statistical significance. The relatively low effect sizes indicate that, while the differences between groups were significant, their practical significance may be limited, highlighting the need to consider other unmeasured factors.^15^ This suggests that while group identity is a meaningful factor in understanding mental health patterns, a substantial portion of the variability in symptoms is attributable to individual‐level factors. Therefore, counseling approaches should not only be culturally informed, considering group‐level trends, but also be highly individualized to address each student's unique needs and circumstances.

### Limitations and directions for future research

This study has several limitations. First, the imbalance in sample sizes across racial and religious groups may have affected the robustness of the results. Second, the use of broad identity categories (e.g., “Asian American”) may obscure within‐group differences that could yield more nuanced insights. Third, the single‐institution design restricts the generalizability of these findings to other university contexts, and the cross‐sectional nature of the data prevents causal inference. Fourth, we pooled data collected before, during, and after the COVID‐19 pandemic, a period known to have substantially affected young adults' mental health, which may have introduced temporal confounding.^8^, ^16^ Future research should address these limitations by recruiting more balanced samples, incorporating finer‐grained identity measures, using multi‐institutional and longitudinal designs, and accounting for temporal effects. Additionally, examining the interaction effects between race and religious identity (e.g., via two‐way MANOVA) could extend the current findings and provide deeper insights into intersectional influences on student mental health. Collectively, these would help develop interventions that are both culturally informed, acknowledging group‐level trends, and highly individualized, responsive to each student's unique experiences and needs.

## CONCLUSION

This study revealed significant differences in mental health conditions among university students who have accessed campus counseling services, demonstrating that psychological symptoms vary considerably between racial and religious subgroups. The findings showed that specific groups (Asian American, Multiracial, Hindu, and Buddhist students) experience heightened levels of distress, while others (White and Jewish students) report lower levels of certain symptoms. These differences underscore the importance of recognizing the diverse mental health needs of student populations and tailoring support services accordingly.^17^ By highlighting these variations, this study contributes to a deeper understanding of the complex factors that influence mental health among university students and emphasizes the need for targeted, culturally sensitive interventions within campus counseling services.^14^

## AUTHOR CONTRIBUTIONS

*Conceptualization*: Ryo Horita. *Data curation*: Ryo Horita. *Formal analysis*: Nicole Nagib. *Supervision*: Mayumi Yamamoto. *Writing—original draft*: Nicole Nagib, Ryo Horita, and Natalie Nagib. *Writing—review and editing*: Nicole Nagib, Ryo Horita, Natalie Nagib, Taku Fukao, Nanako Imamura, Satoko Tajirika, Miho Adachi, Minako Kawamoto, and Mayumi Yamamoto.

## CONFLICT OF INTEREST STATEMENT

The authors declare no conflicts of interest.

## ETHICS APPROVAL STATEMENT

The study design was approved by the ethical review committee of the Graduate School of Medicine, Gifu University, Japan (no. 2021‐B114).

## PATIENT CONSENT STATEMENT

Written informed consent was obtained from all participants in the study.

## CLINICAL TRIAL REGISTRATION

N/A.

## Data Availability

The data that support the findings of this study are available on request from the corresponding author. The data are not publicly available due to privacy or ethical restrictions. All relevant data are included in the paper.
